# Gastric Adenocarcinomas with CDX2 Induction Show Higher Frequency of *TP53* and *KMT2B* Mutations and *MYC* Amplifications but Similar Survival Compared with Cancers with No CDX2 Induction

**DOI:** 10.3390/jcm13247635

**Published:** 2024-12-15

**Authors:** Ioannis A. Voutsadakis

**Affiliations:** 1 Algoma District Cancer Program, Sault Area Hospital, 750 Great Northern Road, Sault Ste Marie, ON P6B 0A8, Canada; ivoutsadakis@yahoo.com or ivoutsadakis@nosm.ca; 2 Division of Clinical Sciences, Section of Internal Medicine, Northern Ontario School of Medicine, Sudbury, ON P3E 2C6, Canada

**Keywords:** stomach cancer, transcription factor, induced expression, CDX2, HNF4A, SOX2, The Cancer Genome Atlas

## Abstract

**Background**: Gastric cancer is one of the most prevalent gastrointestinal cancers. Mortality is high, and improved treatments are needed. A better understanding of the pathophysiology of the disease and discovery of biomarkers for targeted therapies are paramount for therapeutic progress. CDX2, a transcription factor of hindgut specification, is induced in several gastric cancers, especially with intestinal differentiation, and could be helpful for defining sub-types with particular characteristics. **Methods**: Gastric cancers with induced CDX2 mRNA expression were identified from the gastric cohort of The Cancer Genome Atlas (TCGA) and were compared with cancers that had no CDX2 mRNA induction. Induced CDX2 mRNA expression was defined as mRNA expression z-score relative to all samples above 0, and non-induced CDX2 mRNA expression was defined as mRNA expression z-score relative to all samples below −1. **Results**: Patients with gastric cancers with CDX2 mRNA induction were older, had less frequently diffuse histology, and more often had mutations in *TP53* and *KMT2B* and amplifications in *MYC*. CDX2 induction was correlated with HNF4α induction and was reversely correlated with SOX2. Gastric cancers with CDX2 mRNA induction showed lower PD-L1 expression than cancers with lower CDX2 expression but did not differ in CLDN18 mRNA expression. Progression-free and overall survival of the two groups was also not significantly different. **Conclusion**: Gastric cancers with CDX2 mRNA induction displayed specific characteristics that differentiate them from cancers with no CDX2 induction and could be of interest for optimizing current and future therapies.

## 1. Introduction

Gastric adenocarcinoma represents an aggressive malignancy with high prevalence in parts of the world [1]. Gastric cancer constitutes the fifth most prevalent type of cancer worldwide, with almost a million new cases annually [2]. Despite improvements in treatment, metastatic disease remains highly lethal, and therapies are only moderately effective. Chemotherapy remains the backbone of systemic treatment for metastatic disease, with targeted treatments providing moderate incremental benefits in sub-sets of patients [3]. For example, therapeutic targeting of the EGFR family receptor kinase HER2 has improved outcomes of patients with tumors that show over-expression or amplification of this receptor [4,5]. An arising target for monoclonal antibody therapy, in conjunction with chemotherapy, is the adhesion molecule claudin 18.2, which is frequently expressed in gastric cancers [6]. Results from two randomized trials have confirmed improved survival with the addition of the claudin 18.2 targeting monoclonal antibody zolbetuximab to fluoropyrimidine-based chemotherapy in gastric cancer [7,8]. Immunotherapy with immune checkpoint inhibitors has also provided benefit in gastric cancer patients with expression of the target ligand PD-L1 [9,10]. Moreover, gastric cancers with mismatch repair defects or high tumor mutation burden may derive benefit from immunotherapy [11,12]. These targeted therapies, although they provide in most cases only small survival benefits, represent the proof of principle paving the way for additional therapies based on rational targeting, with the hope for longer-lasting results in a greater percentage of patients.

The premise of targeted therapies is the identification and validation of biomarkers that determine the appropriate sub-sets of cancers with the highest probability of response [13]. Therefore, classification of gastric cancers in groups with particular characteristics and molecular alterations is important for therapeutic progress. For example, the benefit of zolbetuximab in combination with chemotherapy was observed in cancers with moderate or strong expression of the target claudin 18.2 in more than 75% of tumor cells, and HER2-targeted therapies are of benefit in cancers with at least some degree of expression of the target receptor [5,7].

The two main histologic sub-types of gastric cancers, the intestinal sub-type and the diffuse sub-type have well-defined differences in molecular alterations and clinical behavior [14]. However, these have not been translated to specific therapies. The diffuse sub-type is characterized by frequent loss of expression of the adhesion molecule and tumor suppressor E-cadherin. Tumor suppressor loss of function is notoriously difficult to target as a cancer therapeutic strategy, given that restoration of a defective protein function would mechanistically require repair of its structure. The intestinal type of gastric cancer, similar to Barrett’s esophagus-associated adenocarcinomas of the gastroesophageal junction, is associated with intestinal metaplasia following injury due to inflammation caused by *Helicobacter pylori*, the acidic environment, or bile acids [15,16]. Exposure to these insults induces transcription factor CDX2 (Caudal Homeobox 2), which specifies an intestinal fate and is expressed in low levels in the normal gastric epithelium [17]. CDX2 antagonizes the actions of the transcription factor SOX2 (SRY-related HMG-box transcription factor 2), which normally activates the foregut transcription program, despite the latter still being expressed in metaplastic epithelia [18]. The metaplastic intestinal program associated with CDX2 up-regulation in the stomach increases the risk for the acquisition of additional molecular alterations that lead to intestinal-type gastric cancers [19,20,21]. Therefore, signals that favor the inappropriate induction of CDX2 expression in gastric epithelia may be early harbingers of carcinogenesis and may represent targetable therapeutic opportunities. Given the high prevalence of gastric cancer and the pervading lethality of metastatic gastric cancer, new targeted therapies to supplement the limited number of currently available targeted treatments for the disease constitute an urgent unmet need.

In this investigation, the clinical and genomic profile of gastric cancers with CDX2 up-regulation is compared with the profile of gastric cancers with suppressed CDX2 expression. The expression of biomarkers with therapeutic implications in the two groups is also examined.

## 2. Methods

The Cancer Genome Atlas (TCGA) gastric cancer cohort consists of 440 patients and provides de-identified patient-level information regarding clinical and genomic tumor characteristics, which are publicly available [22]. Genomic analyses in TCGA were performed with a whole exome next-generation sequencing platform. TCGA has generated data on mutations, copy number alterations, and structural variants. It also provides information on mRNA expression. For single nucleotide mutation calling, various pipelines were used by the institutions that provided genomic data [23]. TCGA used the RSEM (RNA-Seq by expectation–maximization) algorithm for normalization of mRNA expression from RNA-Seq data [24]. An advantage of this algorithm is that it does not require reference genome data. In addition, TCGA provided an aneuploidy score (AS) as a measure of chromosomal instability. The score is calculated by the summation of the number of chromosome arms in each sample that have copy number alterations (gains or losses). A chromosome arm was considered copy number altered, gained, or lost for the AS calculation if there was a somatic copy number alteration in more than 80% of the length of the arm as calculated by the ABSOLUTE algorithm from Affymetrix 6.0 SNP arrays [25]. Chromosome arms with somatic copy number alterations in 20% to 80% of the arm length were considered indeterminate. Chromosome arms with somatic copy number alterations in less than 20% of the arm length were considered not altered.

All primary analyses were performed based on data contained in the cBioportal for Cancer Genomics site (www.cbioportal.org, accessed on 11 October 2024) [26,27]. This user-friendly site provides clinical and genomic data from a variety of source studies, including the hallmark studies of TCGA and other investigator groups. cBioportal necessitates minimal bioinformatics expertise for access and navigation [26,27]. The groups of interest with normal CDX2 mRNA suppression and CDX2-induced expression were constructed from the TCGA gastric cancer cohort through cBioportal, using the “mRNA expression z-scores relative to all samples” and the “download altered” functionalities. The normally suppressed CDX2 mRNA group was defined as having mRNA expression z-score relative to all samples below −1, and the group with induced expression of CDX2 was defined as having mRNA expression z-score relative to all samples above 0.

The statistical analysis was performed with Fisher’s exact test or the x^2^ test for categorical variables and with the *t* test for continuous variables. Correlations of expressions of genes of interest were calculated with the Pearson’s correlation coefficient. Survival analyses were performed with the construction of Kaplan–Meier curves. The log-rank test was used to compare Kaplan–Meier survival curves. All statistical comparisons were considered significant at the level of *p* < 0.05.

## 3. Results

The group of gastric cancer patients with induced CDX2, defined as mRNA expression z-score relative to all samples above 0, consisted of 240 patients (54.5% of the patients of the entire cohort) in the TCGA cohort. The group with suppressed CDX2 expression, defined as mRNA expression z-score relative to all samples below −1, consisted of 62 patients (14.1%). Gastric cancer patients with higher CDX2 expression were older (mean age 66.5 years old) than patients with suppressed CDX2 expression (mean age 61.9 years old, Student’s *t* test *p* < 0.001, Table 1 and Supplemental Table S1). In addition, a trend for fewer patients with early-onset gastric cancer (diagnosed at age younger than 50 years old) at presentation was observed in the group with induced CDX2 expression (Fisher’s exact test *p* = 0.09, Table 1). Diffuse histology and high grade were less prevalent in gastric cancers with higher CDX2 expression (χ^2^ test *p* = 0.02 and Fisher’s exact test *p* = 0.0004, respectively, Table 1). In contrast, the two groups did not differ in the stage of the disease at presentation. There were also no differences in the proportion of patients in the two groups with high TMB and high chromosome instability by AS and FGA scores (Table 2 and Supplemental Table S2). The group with low CDX2 expression had a significant proportion of patients belonging to the EBV-associated genomic category (29.3% versus 1.4% in the group with induced CDX2).

**Figure 1:**
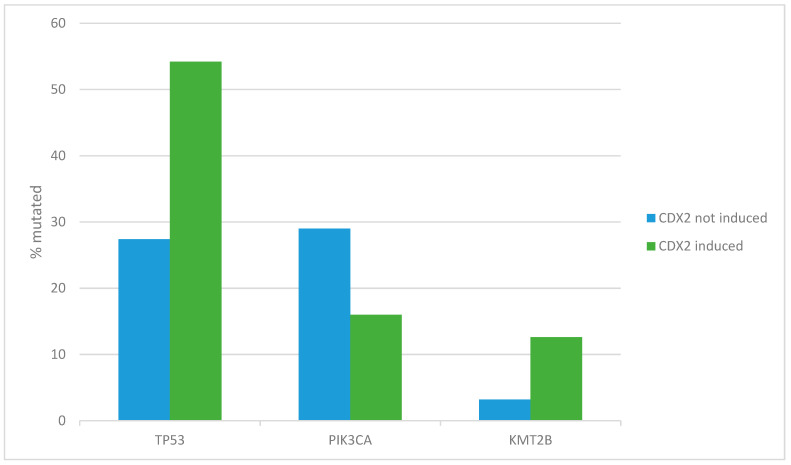
Prevalence of the most frequent gastric cancer mutations in patients with no CDX2 induction (mRNA expression z-score relative to all samples < −1) and with CDX2 induction (mRNA expression z-score relative to all samples > 0). The prevalence of *TP53* and *KMT2B* mutations was higher in gastric cancers with CDX2 induction (54.2% and 12.6% versus 27.4% and 3.2% in cancers with low CDX2, Fisher’s exact test *p* = 0.0002 and *p* = 0.03, respectively). The prevalence of *PIK3CA* mutations was higher in gastric cancers with no CDX2 induction (29% versus 16% in cancers with CDX2 induction, Fisher’s exact test *p* = 0.02).

The most frequently mutated tumor suppressor gene in cancer, *TP53*, showed a higher mutation prevalence in gastric cancers with high CDX2 expression (54.2% versus 27.4% in cancers with low CDX2, Fisher’s exact test *p* = 0.0002, Figure 1). Among other gastric cancer-associated genes, the epigenetic modifier *KMT2B* was also more frequently mutated in cancers with higher CDX2 expression (12.6% versus 3.2% in cancers with lower CDX2, Fisher’s exact test *p* = 0.03, Figure 1). In contrast, the gene encoding for the alpha catalytic sub-unit of kinase PI3K, PIK3CA, was more frequently mutated in gastric cancers with lower expression of CDX2 (29% versus 16% in cancers with higher CDX2, Fisher’s exact test *p* = 0.02, Figure 1).

Mutation frequency in other DNA damage repair (DDR)-related genes, other epigenetic modifiers, or genes encoding receptor tyrosine kinases did not differ significantly between the two groups (Table 3, Table 4 and Table 5 and Supplemental Tables S3–S5). The two most frequently mutated DDR genes in gastric cancer, *ATM* and *BRCA2*, with overall mutation prevalence of 9.6% and 8.7%, respectively, in the gastric cohort of TCGA, were mutated in 10.1% and 7.6% of higher CDX2-expressing cancers and in 9.7% and 8.1% of CDX2-lower-expressing cancers (Fisher’s exact test *p* = 1 for both comparisons, Table 3).

The two most frequently mutated epigenetic modifier genes, *ARID1A* and *KMT2D*, with overall mutation prevalence of 25.2% and 16.7%, respectively, in TCGA, were mutated in 24.8% and 16% of higher CDX2-expressing cancers and in 30.6% and 21% of CDX2-lower-expressing cancers (Fisher’s exact test *p* = 0.41 and 0.34, respectively, Table 4).

**Figure 2:**
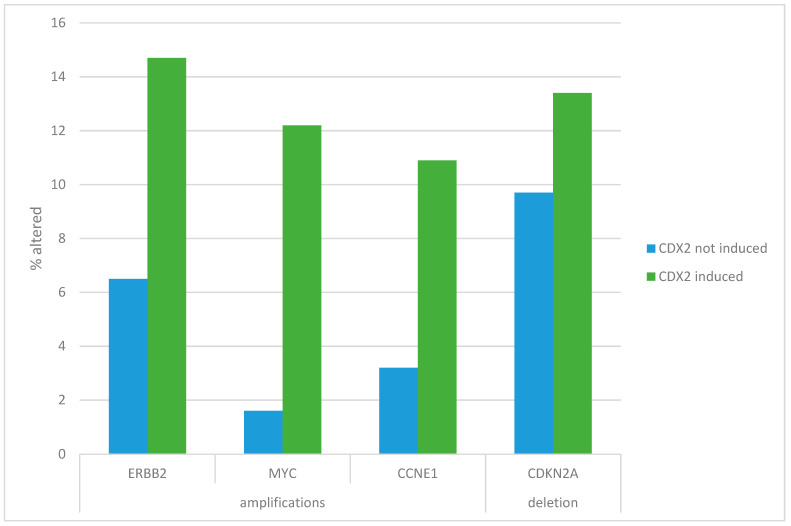
Prevalence of the most frequent gastric cancer copy number alterations in patients with no CDX2 induction (mRNA expression z-score relative to all samples < −1) and with CDX2 induction (mRNA expression z-score relative to all samples > 0). The prevalence of amplifications in *MYC* was higher in gastric cancer with CDX2 induction (12.2%) than in cancers without CDX2 induction (1.6%, Fisher’s exact test *p* = 0.008). The prevalence of amplifications in *ERBB2* showed a statistically non-significant trend for higher expression in gastric cancer with CDX2 induction (14.7%) than in cancers without CDX2 induction (6.5%, Fisher’s exact test *p* = 0.09). Similarly, the prevalence of amplifications in *CCNE1* showed a statistically non-significant trend for higher expression in gastric cancer with CDX2 induction (10.9%) than in cancers without CDX2 induction (3.2%, Fisher’s exact test *p* = 0.08). The prevalence of deletions of CDKN2A was not significantly different in gastric cancers with CDX2 induction (13.4%) and in cancers without CDX2 induction (9.7%, Fisher’s exact test *p* = 0.52).

The most frequently amplified oncogenes in gastric cancers, including *MYC*, *ERBB2* (encoding for HER2), and *CCNE1* (encoding for cyclin E), showed higher prevalence in the higher CDX2-expressing group, which was statistically significant for *MYC* (Fisher’s exact test *p* = 0.008, Figure 2). *MYC*-amplified cases represented 12.2% of CDX2 high-expressing cases and 1.6% of CDX2 lower expressors (Figure 2). *ERBB2* amplifications also showed a trend for higher prevalence in the higher CDX2-expressing group (14.7% versus 6.5% in the CDX2-lower-expressing group, Fisher’s exact test *p* = 0.09, Figure 2). Similarly, a trend was observed for *CCNE1* (10.9% in the CDX2-higher-expressing group versus 3.2% in the CDX2-lower-expressing group, Fisher’s exact test *p* = 0.08, Figure 2). In contrast, the most frequent deletion in gastric cancers at chromosome 9p21.3, encompassing the locus of tumor suppressor *CDKN2A*, had similar prevalence in the two groups (Fisher’s exact test *p* = 0.52, Figure 2).

**Figure 3:**
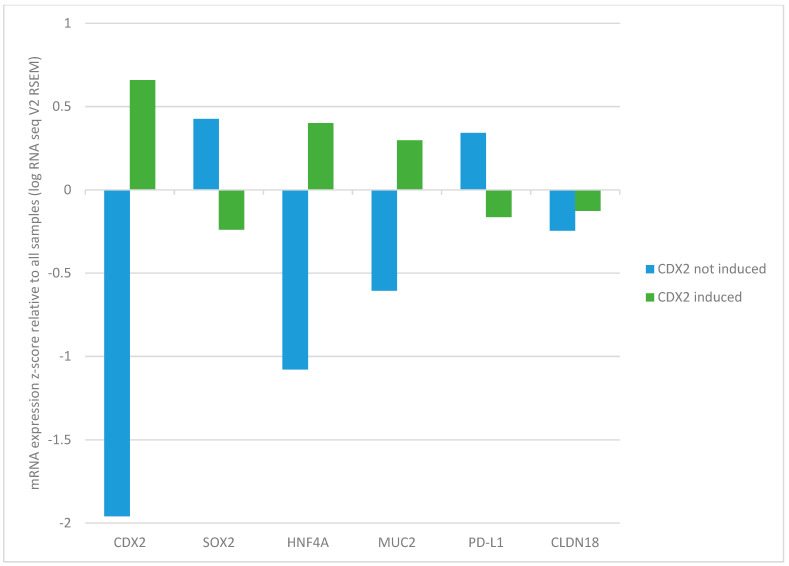
mRNA expression of genes in the CDX2 network and clinically relevant biomarkers in patients with no CDX2 induction (mRNA expression z-score relative to all samples < −1) and with CDX2 induction (mRNA expression z-score relative to all samples > 0). mRNA expression of HNF4A and MUC2 was significantly higher in gastric cancers with CDX2 induction (Student’s *t* test *p*< 0.0001 for both comparisons). mRNA expression of SOX2 and PD-L1 was significantly higher in gastric cancers without CDX2 induction (Student’s *t* test *p*< 0.0001 and *p* = 0.0003, respectively). mRNA expression of CLDN18 was not significantly different between the two groups (Student’s *t* test *p* = 0.44).

The group of CDX2-induced gastric cancers showed a parallel up-regulation of transcription factor HNF4A (mean expression z-score relative to all samples = 0.4), while the transcription factor SOX2, an inducer of foregut specification, was down-regulated (mean expression z-score relative to all samples = −0.23, Figure 3). In contrast, cancers with low CDX2 showed higher SOX2 expression (mean expression z-score relative to all samples = 0.42, Student’s *t* test *p* < 0.0001) and significant HNF4A suppression (mean expression z-score relative to all samples = −1.07, Student’s *t* test *p* < 0.0001, Figure 3). Gastric cancers with CDX2 induction and a parallel induction of HNF4A (defined as mRNA expression z-score relative to all samples above 1) showed an even lower percentage of diffuse histology (3.1%) compared with all cancers with CDX2 induction (diffuse histology in 11.2% of cases, Table 6) or the cohort with no CDX2 induction (diffuse histology in 24.2%).

In addition, gastric cancers with CDX2 induction and a parallel induction of HNF4A displayed a higher rate of CIN (83% of cases) compared with all cancers with CDX2 induction (CIN in 63.3% of cases, Table 7) or the cohort with no CDX2 induction (CIN in 43.1% of cases). On the other hand, the group of gastric cancers with CDX2 and HNF4A induction had lower prevalence of MSI and no cases with EBV association (Table 7). *TP53* mutations and *ERBB2* amplifications were more prevalent (67.2% and 23.4%, respectively) in gastric cancers with CDX2 induction and a parallel induction of HNF4A than in the whole group of cancers with CDX2 induction (54.2% and 14.7%, respectively).

The mucin target of CDX2, mucin 2, was up-regulated in the group with CDX2 induction (mean expression z-score relative to all samples = 0.29 versus mean expression z-score relative to all samples = −0.6 in the group with low CDX2, Student’s *t* test *p* < 0.0001, Figure 3). PD-L1 immune checkpoint ligand is a biomarker used to predict response to checkpoint inhibitor immunotherapy in gastric cancers. PD-L1 mRNA expression was lower in gastric cancers with increased CDX2 expression compared with gastric cancers with low CDX2 expression (Student’s *t* test *p* < 0.0001, Figure 3).

Claudin 18.2 is a gastric-specific isoform of the claudin group of adhesion proteins. Fusions of the *CLDN18* gene encoding for claudin 18 with various partner genes, most commonly with *ARHGAP26*, were observed in 2.8% of patients in the gastric TCGA cohort and had a similar prevalence in cases with low and high CDX2 expression (3.2% and 3.4%, respectively; Fisher’s exact test *p* = 1). The mean expression of claudin 18 mRNA did not differ significantly between the CDX2 groups with low and high CDX2 expression (Student’s *t* test *p* = 0.44, Figure 3).

**Figure 4:**
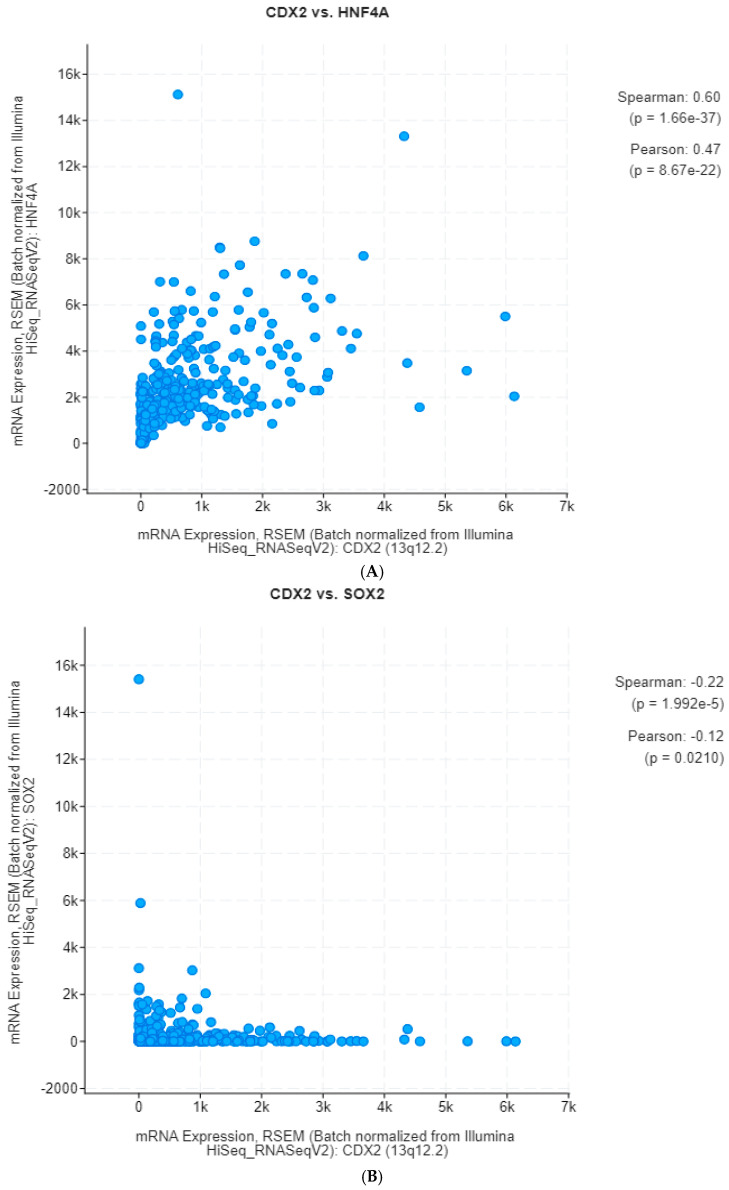

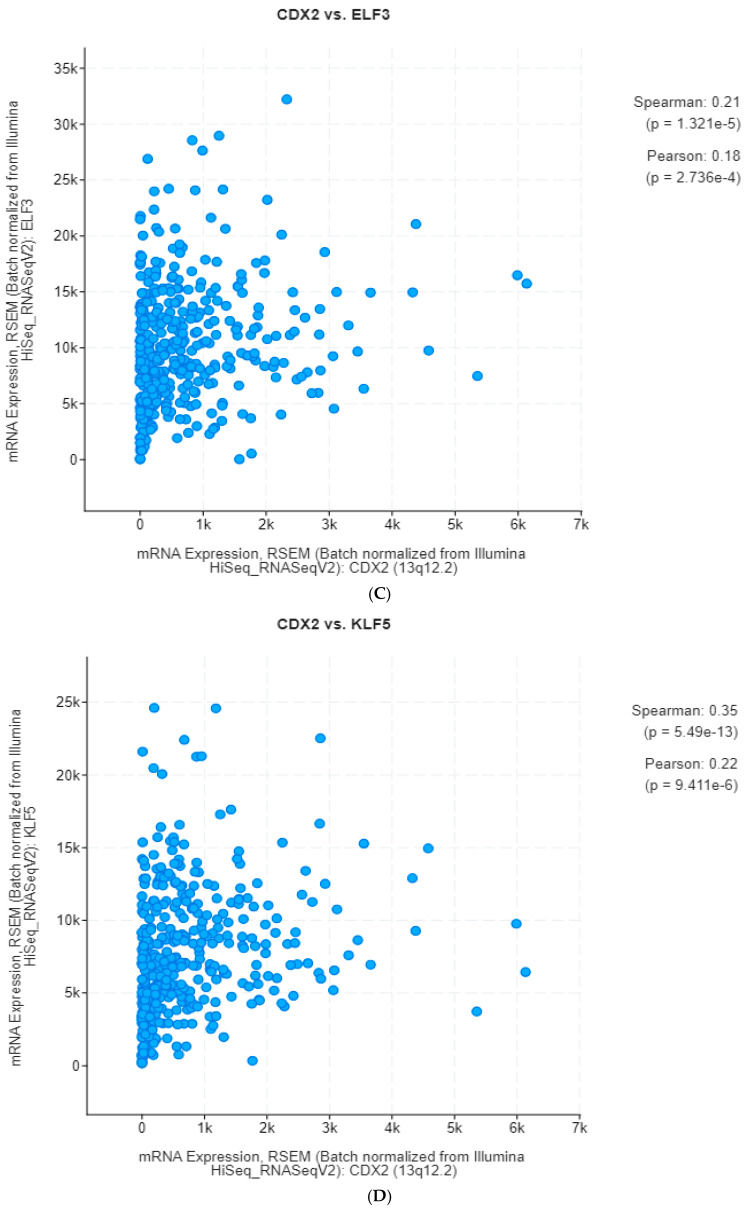
Correlations of CDX2 expression with the expression of other gastrointestinal fate-determining transcription factors. (**A**) Correlation with the expression of HNF4A. (**B**) Correlation with the expression of SOX2. (**C**) Correlation with the expression of ELF3. (**D**) Correlation with the expression of KLF5.

CDX2 mRNA expression showed a strong correlation with the mRNA expression of transcription factor HNF4α (Spearman correlation coefficient 0.60, *p* = 1.66 × 10^−37^, Figure 4A). In contrast, mRNA expression of transcription factor SOX2 displayed a reverse correlation with CDX2 mRNA expression (Spearman correlation coefficient −0.22, *p* = 1.99 x 10^−5^, Figure 4B). The expression of two other transcription factors, ELF3 and KLF5, also correlated with the expression of CDX2 in gastric cancer (Spearman correlation coefficient 0.21, *p* = 1.07 × 10^−5^, and Spearman correlation coefficient 5.49, *p* = 1.07 × 10^−13^, respectively, Figure 4C and Figure 4D).

**Figure 5:**
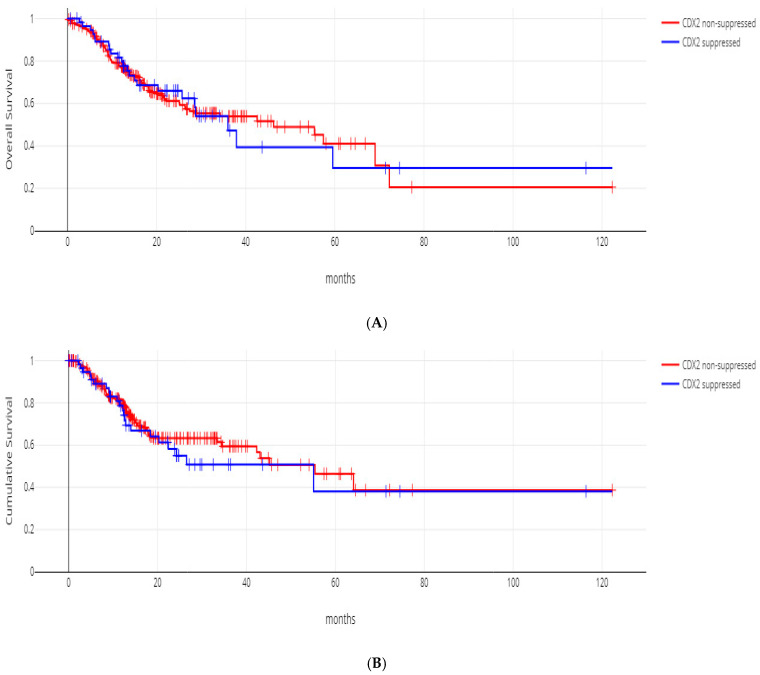
(**A**) OS of patients with induced CDX2 gastric cancers versus patients with no CDX2 induction, log-rank *p* = 0.94. (**B**). PFS of patients with induced CDX2 gastric cancers versus patients with no CDX2 induction, log-rank *p* = 0.49.

The progression-free survival (PFS) and overall survival (OS) of the two groups with CDX2 mRNA induction and no CDX2 induction did not differ significantly (log-rank *p* = 0.49 and 0.94, respectively, Figure 5A and Figure 5B).

## 4. Discussion

The homeodomain transcription factor CDX2 has a role in normal development, participating in the midgut and hindgut specification and intestinal gene expression [28]. In contrast, CDX2 is not expressed in the foregut and the nascent stomach, and the role of foregut specification is fulfilled by transcription factor SOX2 expression and function [29]. Ectopic cdx2 expression in the stomach induced gastric intestinal metaplasia in transgenic mice, and CDX2 is induced in intestinal gastric metaplasia and gastric carcinomas [30,31,32]. A significant percentage of gastric cancers, albeit varying according to the criteria of positivity used and the population studied, express CDX2. In a series of 80 gastric cancer patients from the Middle East, 38.7% of gastric cancers expressed CDX2, defined as at least 10% of cancer cells with nuclear staining in immunohistochemistry sections [32]. CDX2 positivity was more commonly observed in older patients, but there were no significant associations with gender, grade, stage, or Lauren histologic type [32]. In a series of 92 localized gastric cancer patients from Spain, who underwent surgical resection, 68.5% of tumors were CDX2 positive [33]. In this study, CDX2 positivity was defined as moderate nuclear staining in at least 5% of tumor cells. Stage 1 tumors were more frequently CDX2 positive, and CDX2 positivity was associated with improved survival in this study [33]. In a series of 44 patients from the United States, CDX2 positivity defined as nuclear staining in 25% or more of tumor cells was present in 59.1% of cases [34]. CDX2 protein positivity was associated with better prognosis in EBV-negative, MMR-proficient gastric cancers in a series of 1158 advanced gastric cancer patients [35]. The study used tissue microarrays and considered positive samples with any intensity and percentage of positivity, even if these were not stronger than surrounding normal gastric mucosa. Other studies have confirmed the expression of CDX2 protein in subsets of gastric cancers and the concomitant expression of tumor suppressor p53, denoting mutated status [36,37].

In the research presented herein, gastric cancers with CDX2 mRNA induction, representing 54.5% of the gastric cancers from the extensive TCGA genomic cohort of 440 patients, had an older mean age of onset and were more often of intestinal or mixed histology than gastric cancers with normally suppressed CDX2 expression. In addition, a higher percentage of cancers with CDX2 mRNA induction showed CIN and MSI high status, while association with EBV was rare. Mutations in the tumor suppressor *TP53*, an alteration commonly associated with drug resistance, were significantly more frequent in gastric cancers with CDX2 mRNA induction. However, despite this, gastric cancers with CDX2 mRNA induction had a higher frequency of low grade, and survival outcomes did not differ between the two groups. The two groups did differ in the prevalence of *ERBB2* amplifications, which were more frequent in the group with CDX2 mRNA induction and in PD-L1 expression, which was higher in the group with normally suppressed CDX2 expression, but did not differ in CLDN18 expression. These differences have therapeutic repercussions, as targeted therapies and immunotherapies are available for sub-sets of gastric cancers with these alterations. For example, monoclonal antibodies and antibody–drug conjugates targeting Claudin 18.2 would potentially be effective for subsets of patients in both groups [38]. Similarly to the findings from TCGA, in a series of intestinal type gastric cancers, HER2 over-expression was observed more often (50% of cases) in CDX2-positive cases than in CDX2-negative cases, where HER2 over-expression was only present in 1 of 15 (6.7%) of patients (*p* = 0.009) [34]. In contrast, PD-L1 expression was not different according to CDX2 status in this study. The discordant result compared to TCGA may be due to the fact that the study employed IHC to determine the CDX2 and PD-L1 protein expression status, while TCGA results were obtained from mRNA expression levels. Besides ERBB2, other genes encoding receptor tyrosine kinases or downstream proteins of their pathways are recurrently amplified, albeit in lower frequencies, in gastrointestinal cancers, such as gastric, esophageal, and colorectal carcinomas [39]. These include receptors EGFR, FGFR1, FGFR2, and IGF1R; and MET, ligands FGF3, FGF4, and FGF19; and VEGFA and intracellular transducers KRAS and GAB2. Recurrent amplifications in the receptor tyrosine kinase-initiated pathways bear witness to the oncogenic function of the transduction signals propagated by these cascades, which favor clones that have acquired increased gene dosage in their components but also provide targets for therapeutic manipulation.

The induction of CDX2 expression may be due to aberrant hypomethylation of CpG islands surrounding the transcription-initiating site at the 5-UTR of the gene [40]. CDX2 expression induction in gastric intestinal metaplasia is independent of the presence of gastric infection with *Helicobacter pylori* (*H. pylori*), suggesting that other inflammatory metaplasia-promoting insults may also induce this transcription factor [41]. In agreement with this suggestion, gastric intestinal dysplasia has been described in patients without *H. pylori* infection [42]. In diffuse gastric cancer, absence of *H. pylori* infection was associated with a purely gastric phenotype and absence of CDX2 expression, while *H. pylori*-associated diffuse cancers displayed more often CDX2 expression and co-expressed intestinal proteins such as mucin 2 and CD10 [43]. *H. pylori*-associated and *H. pylori*-naïve diffuse gastric cancers also differed in specific chromosome arm alterations, with gains in 8p, 8q, 7p, and 7q, as well as losses in 16q being more common in *h. pylori*-infected cases. Bile acid exposure is another mechanism inducing gastric intestinal metaplasia [44]. The mechanism involves activation of the farnesoid nuclear receptor FXR and the NF-κB pathway, resulting in CDX2 induction [45,46]. Another inflammation-related signaling cascade inducing CDX2 in gastric cancer cells is the interleukin 6 (IL6) cascade, which activates JAK/STAT and ERK/MEK signaling [47].

CDX2 up-regulated the expression of receptor tyrosine kinase EphB2 and reduced the migration of gastric cancer cells [48]. In patient samples, EphB2 expression correlated with CDX2 expression and was increased in gastric intestinal metaplasia and adenocarcinoma tissues compared to normal gastric mucosa. Nevertheless, EphB2-positive gastric cancers had a better prognosis than EphB2-negative cancers. Other established target genes of CDX2, such as MUC2, are upregulated by CDX2 in gastric cancers, as confirmed previously and herein [49].

A master regulator of the endoderm intestinal differentiation, HNF4α showed a strong co-expression with CDX2 at the mRNA level in the TCGA gastric cancer cohort. HNF4α (also known as NR2A1), a transcription factor of the nuclear receptor superfamily, is expressed in the liver and the epithelium of gastrointestinal organs [50]. Early embryonic lethality is observed in hnf4a knockout mice, arguing for the central developmental role of the transcription factor [51]. The nuclear receptor functions in epithelial integrity and protection against microbial-induced inflammation. Linoleic acid has been identified as an endogenous ligand for HNF4A, which, however, does not affect the transcriptional activity of the receptor [52]. HNF4α is upregulated in gastric carcinoma tissues, compared to normal gastric mucosa [53]. HNF4α expression in intestinal-type gastric adenocarcinomas correlated with the expression of MUC2 [54]. Consistent with the current results in gastric adenocarcinomas, gastric intestinal metaplasia and Barrett’s esophagus showed increased expression of HNF4α in conjunction with CDX2 [55]. HNF4α was prominently involved in a group of master transcription factors regulating the epigenome of gastrointestinal cancers [56]. These included also KLF5, ELF3, GATA4, GATA6, another nuclear receptor family member, HNF1α, and transcription factors inducing the interleukin pathway [56]. The promoter of HNF4α was positively regulated by KLF5, ELF3, GATA4, and GATA6, as well as HNF4α itself in a positive feed-forward loop. HNF4α, then, promoted HNF1α expression and the interleukin pathway and resulted in a tumor growth effect.

The Krüppel-like factor 5 (KLF5) is a transcription factor whose expression also correlated with CDX2 expression. KLF transcription factors comprise a family with 17 members [57]. In development, KLF5, together with another KLF family member, KLF4, is involved in pluripotency maintenance through suppression of differentiation to mesoderm and endoderm [58]. KLF4 is also one of the four original transcription factors, together with OCT4, SOX2, and MYC, that can reprogram differentiated cells to induced pluripotent stem cells [59,60]. KLF5 was expressed by immunohistochemistry in 45.7% of patients with localized gastric cancer undergoing curative gastrectomies in a Korean center, and this expression was associated with a trend towards better 5-year survival [61].

In contrast to HNF4α and KLF5, the expression of the other master pluripotency transcription factor, SOX2, anti-correlated with the expression of CDX2. SOX2 is a regulator of foregut development and has been associated with both tumor-promoting and tumor-curtailing effects in gastric cancer [62]. Over-expression of SOX2 promotes the cancer stem cell phenotype denoted by self-renewal, metastasis, and drug resistance [62]. On the other hand, SOX2 interferes with the function of CDX2 and prevents intestinal metaplasia [63]. Therefore, the interplay of the two transcription factors preserves gastric epithelial homeostasis and prevents cancer arising from excessive stem cell activity or intestinal metaplasia. In contrast, deregulation of this fine balance promotes carcinogenesis through deregulated stem cell activation or intestinal metaplasia development.

A signaling pathway that regulates CDX2 in embryogenesis and may play a role in CDX2 deregulation in gastric carcinogenesis is the Hippo pathway [64,65,66]. Hippo is a pathway conserved from drosophila to mammals and is an important regulator of tissue homeostasis and organ size control [67]. In early mammalian embryonic development, CDX2 expression specifies the trophectoderm and is induced under the influence of the hippo pathway effector transcription factor TEAD4 and the NOTCH pathway, while transcription factors OCT4, SOX2, and NANOG specify the inner cell mass that will give rise to the embryo and the yolk sac [64]. A similar paradigm with the balance between CDX2 and SOX2 in the specification of upper and lower gastrointestinal epithelia suggests that programs that are normally functional in normal embryogenesis are aberrantly reactivated in gastric carcinogenesis. The transcription factors regulated by the Hippo pathway promote epithelial to mesenchymal transition (EMT) and stemness in co-operation with the TGFβ signaling pathway and induction of CTCF, AXL, and core EMT factors Snail and Slug [68]. In gastric cancers, expression of the Hippo pathway transcription factors YAP1 and TEAD was up-regulated in parallel with CDX2, while the negative regulators of the pathway, serine/threonine kinases MST1 and LATS1, were down-regulated, compared with normal gastric epithelia [66]. Similar deregulation of the expression of the components of the Hippo pathway was observed in chronic atrophic gastritis with intestinal metaplasia, suggesting that the activation of the Hippo pathway is an early feature of gastric neoplastic transformation and may contribute to the aberrant CDX2 induction, as is normally observed in embryogenesis. Treatment of gastric cancer cells and human xenografts in mice with ursolic acid activated the kinases MST1, MST2, and LATS1 and inhibited YAP1, leading to decreased proliferation and metastases [69]. The anti-proliferative result was mediated by activation of RASSF1 (Ras association domain family 1), and the effects of ursolic acid on YAP1 were reversed by silencing RASSF1. Interestingly, both CDX2 and HNF4α possess binding sequences in the promoter of YAP1, and a positive feed-forward loop may be operating in gastric cancers with CDX2 induction [70]. The effector transcription factors of the Hippo pathway, YAP/TAZ and TEAD, have been proposed as synthetic lethal with inhibition of the KRAS pathway in cells with aberrant activation due to KRAS G12C mutations [71]. The activity of the Hippo pathway transcription factors substitutes for the activity of oncogenic KRAS G12C in cancer cells treated with inhibitors of KRAS, leading to treatment resistance through activation of the PI3K/AKT cascade and MYC activation [72]. Pharmacologic inhibition of TEAD arises as a therapeutic intervention to reverse KRAS G12C inhibitor resistance [73].

**Figure 6:**
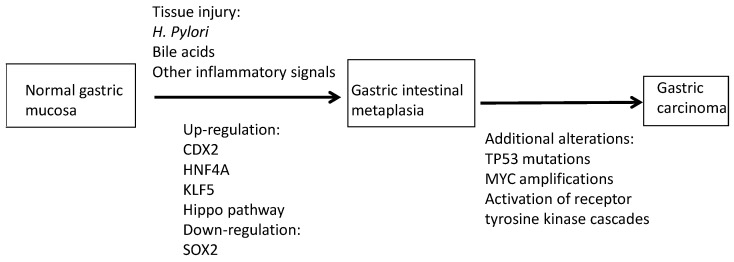
A schematic model of gastric carcinogenesis as presented in the article.

Although deregulations of the developmental fate of gastric epithelial cells in gastric carcinogenesis suggest an involvement of embryonic programs in cancer, a direct cancer-promoting effect may not be present. Instead the deregulation of these programs operating in normal development may be a physiologic response to tissue injury, which then may become the basis for neoplastic transformation if additional alterations accumulate. In this model, gastric epithelial tissue injury promotes chronic inflammation and intestinal metaplasia through induction of CDX2. Molecular events that favor CDX2 induction include developmental programs, such as the activation of the transcription factors regulated by the Hippo pathway (Figure 6). Gastric epithelia that have undergone intestinal metaplasia are prone to neoplastic transformation with ongoing activity of CDX2 and feedforward activation of the Hippo pathway, as suggested by the presence of binding sites of CDX2 in the promoters of the Hippo pathway transcription factors [70]. Additional molecular alterations, including *TP53* mutations, which disable apoptosis, epigenetic deregulation, and *MYC* activation, contribute to the carcinogenesis cascade. These alterations may also contribute to therapy resistance, and although no direct inhibitors of CDX2 exist yet, the network could be targeted for reversal of this resistance. An example, as mentioned above, is the novel pan-TEAD inhibitor, which reverses resistance to KRAS inhibitors [73]. KRAS is altered in a minority of gastric cancers, but other components of its network activated by receptor tyrosine kinases are frequently altered, and combining inhibition of the KRAS/BRAF/MEK and the PI3K/AKT cascades with TEAD inhibitors in specific gastric cancer cases with the appropriate target alterations could be a viable strategy. TEAD inhibitors could also synergize with immunotherapy treatments, given that YAP/TEAD are suppressors of the expression of major histocompatibility complex I (MHC I), which presents antigens to CD8+ cytotoxic T cells [74]. Therefore, inhibiting the pathway may reverse immune evasion and promote immune-mediated tumor control mediated by immune checkpoint inhibitors [75]. Moreover, reversing resistance due to p53 debilitation could also arise as a therapeutic strategy in these cancers, should modulators of the MDM2/p53 pathway reach the clinic, and p53 deficiency may also be a synthetic lethal vulnerability [76]. The prognostic implications of the aberrant metaplastic program resulting from the induction of transcription factors not normally expressed in adult tissues may vary according to the specific subsequent alterations that are favored. Regardless, inhibiting the molecular processes that are at the basis of a subsequent transformation such as CDX2 induction may be a rational therapeutic avenue to explore.

The current analysis has some limitations. The analysis is based on a single genomic cohort, which used several computational pipelines for the calling of the single nucleotide variations. The evaluation was performed exclusively in silico, and no experimental confirmation was included. The source series reported data at the level of DNA and mRNA expression, and no proteomics analysis was available. In addition, the clinical information available was restricted to patient and disease characteristics, and no extensive therapeutic information was available. However, a major strength of the source gastric TCGA cohort, as well as the studies of other cancer cohorts performed by TCGA, is that they have included whole exome sequencing, which allows analysis of genes that are not typically included in the limited targeted sequencing used by other investigators and are publicly available in cBioportal. Data provided by TCGA have consistently been validated in other series and have provided the foundation for clinical development of targeted therapies in various cancers.

Eradication of the *H. pylori* colonizing the gastric mucosa before the induction of intestinal metaplasia, which may lead to non-reversible dysplasia and eventual cancer development, has been the most straightforward therapeutic strategy for prevention of gastric cancer [77]. Intestinal metaplasia may, though, result from other exposures, as discussed above, and therefore targeting it directly would address *H. pylori*-independent pathways. With these considerations in mind, therapies inhibiting the molecular network inducing the metaplastic changes, in which CDX2 plays a key role, would constitute a fundamental advancement in gastric cancer therapeutics.

## Figures and Tables

**Table 1:** Clinical characteristics of the entire TCGA gastric cancer cohort and of the groups with no CDX2 induction (mRNA expression z-score relative to all samples <−1) and with CDX2 induction (mRNA expression z-score relative to all samples >0). Other histologies include signet ring, tubular, papillary, and mucinous. The sum of the CDX2-induced and CDX2-not-induced groups does not equal the number of patients in the entire cohort, as a group of patients did not fulfill the criteria for inclusion in either CDX2 group.

	All (*n* = 440) (%)	CDX2 Not Induced (*n* = 62) (%)	CDX2 Induced (*n* = 240) (%)	*p*-Value
Age (mean)	65.6 ± 10.7	61.9 ± 10.3	66.5 ± 10.3	0.001
Early onset (≤50 years old)				
Yes	35 (8)	8 (12.9)	14 (5.9)	0.09
No	401 (92)	54 (87.1)	223 (94.1)	
NA	4		3	
Sex				
Male	284 (64.5)	45 (72.6)	157 (65.4)	0.36
Female	156 (35.5)	17 (27.4)	83 (34.6)	
Histology				
Intestinal/NOS adenocarcinoma	246 (55.9)	32 (51.6)	132 (55)	0.02
Diffuse	72 (16.4)	15 (24.2)	27 (11.2)	
Other	122 (27.7)	15 (24.2)	81 (33.8)	
Grade				
1–2	168 (39)	13 (21.7)	110 (46.6)	0.0004
3	263 (61)	47 (78.3)	126 (53.4)	
NA	9	2	4	
Stage				
I	58 (13.8)	13 (22)	35 (15.1)	0.29
II	131 (31.1)	14 (23.7)	77 (33.2)	
III	188 (44.7)	25 (42.4)	102 (44)	
IV	44 (10.4)	7 (11.9)	18 (7.7)	
NA	19	3	8	

NA: Not available; NOS: not otherwise specified.

**Table 2:** Genomic characteristics of the entire TCGA (The Cancer Genome Atlas) gastric cohort and of the groups with no CDX2 induction (mRNA expression z-score relative to all samples <−1) and CDX2 induced (mRNA expression z-score relative to all samples >0). The sum of the CDX2-induced and CDX2-not-induced groups does not equal the number of patients in the entire cohort, as a group of patients did not fulfill the criteria for inclusion in either CDX2 group.

	All (*n* = 440) (%)	CDX2 Not Induced (*n* = 62) (%)	CDX2 Induced (*n* = 240) (%)	*p*-Value
Genomic category				
CIN	223 (58.2)	25 (43.1)	138 (63.3)	<0.0001
GS	50 (13.1)	8 (13.8)	25 (11.5)	
MSI	73 (19.1)	8 (13.8)	47 (21.5)	
EBV	30 (7.8)	17 (29.3)	3 (1.4)	
POLE	7 (1.8)	0	5 (2.3)	
NA	57	4	22	
TMB				
High (>10 mutations/Mb)	99 (22.7)	10 (16.1)	59 (24.8)	0.17
Low (≤10 mutations/Mb)	337 (77.3)	52 (83.9)	179 (75.2)	
NA	4		2	
AS				
<4	120 (28.1)	18 (30)	82 (35.1)	0.61
4–24	283 (66.3)	39 (65)	136 (58.1)	
>24	24 (5.6)	3 (5)	16 (6.8)	
NA	13	2	6	
FGA				
<0.08	138 (31.5)	21 (33.9)	63 (26.5)	0.26
>0.08	300 (68.5)	41 (66.1)	175 (73.5)	
NA	2	0	2	

CIN: chromosomal instability, MSI: microsatellite instability, GS: genomically stable, EBV: Epstein–Barr virus, POLE: polymerase epsilon, TMB: tumor mutation burden, AS: aneuploidy score, FGA: fragment genome altered, NA: not available.

**Table 3:** Mutations in DNA damage response (dDR) genes in samples with and without CDX2 mRNA suppression from the TCGA stomach cancer cohort. The sum of the mutations in each gene does not equal the total number of mutations in the whole cohort of patients because patients with intermediate CDX2 mRNA expressions (−1 to 0) were not included in either of the two compared groups, and some patients had more than one mutation.

Gene	All Patients (%)	CDX2 Not Induced (%)	CDX2 Induced (%)	*p*-Value
*BRCA1*	16 (3.7)	3 (4,8)	8 (3.4)	0.7
*BRCA2*	38 (8.7)	5 (8.1)	18 (7.6)	1
*PALB2*	11 (2.5)	0	7 (2.9)	0.35
*RAD51*	2 (0.5)	0	1 (0.4)	1
*RAD51B*	1 (0.2)	0	0	1
*RAD51C*	1 (0.2)	0	1 (0.4)	1
*RAD51D*	2 (0.5)	0	1 (0.4)	1
*RAD50*	9 (2.1)	0	5 (2.1)	0.58
*XRCC2*	4 (0.9)	0	3 (1.3)	1
*ATM*	42 (9.6)	6 (9.7)	24 (10.1)	1
*ATR*	27 (6.2)	5 (8.1)	12 (5)	0.35
*BRIP1*	7 (1.6)	0	4 (1.7)	0.58
*NBN*	16 (3.7)	1 (1.6)	9 (3.8)	0.69
*MRE11*	7 (1.6)	1 (1.6)	5 (2.1)	1
*CHEK1*	6 (1.4)	0	5 (2.1)	0.58
*CHEK2*	8 (1.8)	0	6 (2.5)	0.35
*BARD1*	15 (3.4)	1 (1.6)	12 (5)	0.31
*BAP1*	13 (3)	1 (1.6)	8 (3.4)	0.69
*POLQ*	36 (8.3)	2 (3.2)	19 (8)	0.26
*CDK12*	22 (5)	1 (1.6)	10 (4.2)	0.46

**Table 4:** Mutations in epigenetic modifiers in samples with and without CDX2 mRNA suppression from the TCGA stomach cancer cohort. The sum of the mutations in each gene does not equal the total number of mutations in the whole cohort of patients because patients with intermediate CDX2 mRNA expressions (−1 to 0) were not included in any of the compared groups, and some patients had more than one mutation.

Gene	All Patients (%)	CDX2 Not Induced (%)	CDX2 Induced (%)	*p*-Value
*ARID2*	35 (8)	8 (12.9)	18 (7.6)	0.2
*ARID1A*	110 (25.2)	19 (30.6)	59 (24.8)	0.41
*ARID1B*	29 (6.7)	5 (8.1)	14 (5.9)	0.55
*ARID5B*	16 (3.7)	1 (1.6)	11 (4.6)	0.47
*KMT2C*	61 (14)	12 (19.4)	35 (14.7)	0.43
*KMT2D*	73 (16.7)	13 (21)	38 (16)	0.34
*KMT2A*	42 (9.6)	3 (4.8)	29 (12.2)	0.1
*KMT2B*	42 (9.6)	2 (3.2)	30 (12.6)	0.03
*DNMT3A*	11 (2.6)	2 (3.2)	7 (2.9)	1
*DNMT1*	16 (3.7)	1 (1.6)	9 (3.8)	0.69
*DNMT3B*	14 (3.2)	2 (3.2)	6 (2.5)	0.67
*KDM5C*	21 (4.8)	2 (3.2)	7 (2.9)	1
*KDM6A*	15 (3.4)	1 (1.6)	10 (4.2)	0.47
*KDM5A*	21 (4.8)	3 (4.8)	11 (4.6)	1
*EP300*	22 (5)	2 (3.2)	13 (5.5)	0.74
*CREBBP*	39 (8.9)	4 (6.5)	27 (11.3)	0.35
*SETD2*	19 (4.4)	2 (3.2)	12 (5)	0.74
*SMARCA2*	27 (6.2)	1 (1.6)	14 (5.9)	0.32
*SMARCA4*	25 (5.7)	0	16 (6.7)	0.05
*SMARCB1*	13 (3)	0	8 (3.4)	0.21

**Table 5:** Mutations in receptor tyrosine kinases in samples with and without CDX2 mRNA suppression from the TCGA stomach cancer cohort. The sum of the mutations in each gene does not equal the total number of mutations in the whole cohort of patients because patients with intermediate CDX2 mRNA expressions (−1 to 0) were not included in any of the compared groups, and some patients had more than one mutation.

Gene	All Patients (%)	CDX2 Not Induced (%)	CDX2 Induced (%)	*p*-Value
*EGFR*	21 (4.8)	1 (1.6)	14 (5.9)	0.32
*ERBB2*	25 (5.7)	3 (4.8)	16 (6.7)	1
*ERBB3*	43 (9.9)	4 (6.5)	26 (10.9)	0.35
*ERBB4*	52 (11.9)	5 (8.1)	31 (13)	0.31
*FGFR1*	13 (3)	1 (1.6)	8 (3.4)	0.69
*FGFR2*	17 (3.9)	3 (4.8)	8 (3.4)	0.7
*FGFR3*	10 (2.3)	0	7 (2.9)	0.35
*FGFR4*	14 (3.2)	3 (4.8)	9 (3.8)	0.71
*PDGFRA*	13 (3)	1 (1.6)	9 (3.8)	0.69
*PDGFRB*	14 (3.2)	1 (1.6)	12 (5)	0.31
*NTRK1*	12 (2.8)	3 (4,6)	6 (2.5)	0.39
*NTRK2*	15 (3.4)	0	13 (5.5)	0.07
*NTRK3*	15 (3.4)	2 (3.2)	11 (4.6)	1
*ALK*	19 (4,4)	1 (1.6)	12 (5)	0.31
*ROS1*	28 (6.4)	1 (1.6)	16 (6.7)	0.21
*MET*	7 (1.6)	1 (1.6)	5 (2.1)	1
*RET*	17 (3.9)	0	10 (4.2)	0.22
*INSR*	14 (3.2)	1 (1.6)	9 (3.8)	0.69
*IGF1R*	18 (4.1)	1 (1.6)	13 (5.5)	0.31
*EPHA1*	13 (3)	2 (3.2)	10 (4.2)	1
*EPHB1*	28 (6.4)	4 (6.5)	16 (6.7)	1

**Table 6:** Clinical characteristics of the entire TCGA gastric cancer cohort with CDX2 induction (mRNA expression z-score relative to all samples > 0) and of the groups with additional HNF4A induction (mRNA expression z-score relative to all samples > 1) and with no HNF4A induction (mRNA expression z-score relative to all samples < 1). Other histologies include signet ring, tubular, papillary, and mucinous.

	All CDX2 > 0 (*n* = 240) (%)	CDX2 > 0 and HNF4A > 1 (*n* = 64) (%)	CDX2 > 0 and HNF4A < 1 (*n* = 176) (%)	*p*-Value
Age (mean)	66.5 ± 10.3	67.3 ± 10.1	66.3 ± 10.4	0.5
Early onset (≤50 years old)				
Yes	14 (5.9)	4 (6.5)	10 (5.7)	0.76
No	223 (94.1)	58 (93.5)	165 (94.3)	
NA	3	2	1	
Sex				
Male	157 (65.4)	46 (71.9)	111 (63.1)	0.22
Female	83 (34.6)	18 (28.1)	65 (36.9)	
Histology				
Intestinal/NOS adenocarcinoma	132 (55)	37 (57.8)	95 (54)	0.05
Diffuse	27 (11.2)	2 (3.1)	25 (14.2)	
Other	81 (33.8)	25 (39.1)	56 (31.8)	
Grade				
1–2	110 (46.6)	38 (61.3)	72 (41.4)	0.007
3	126 (53.4)	24 (38.7)	102 (58.6)	
NA	4	2	2	
Stage				
I	35 (15.1)	13 (20.3)	22 (13.1)	0.56
II	77 (33.2)	20 (31.3)	57 (33.9)	
III	102 (44)	27 (42.1)	75 (44.7)	
IV	18 (7.7)	4 (6.3)	14 (8.3)	
NA	8		8	

NA: Not available; NOS: not otherwise specified.

**Table 7:** Genomic characteristics of the entire TCGA gastric cancer cohort with CDX2 induction (mRNA expression z-score relative to all samples > 0) and of the groups with additional HNF4A induction (mRNA expression z-score relative to all samples > 1) and with no HNF4A induction (mRNA expression z-score relative to all samples < 1). Other histologies include signet ring, tubular, papillary, and mucinous.

	All CDX2 > 0 (*n* = 240) (%)	CDX2 > 0 and HNF4A > 1 (*n* = 64) (%)	CDX2 > 0 and HNF4A < 1 (*n* = 176) (%)	*p*-Value
Genomic category				
CIN	138 (63.3)	49 (83)	89 (56)	0.01
GS	25 (11.5)	2 (3.4)	23 (14.4)	
MSI	47 (21.5)	7 (11.9)	40 (25.2)	
EBV	3 (1.4)	0	3 (1.9)	
POLE	5 (2.3)	1 (1.7)	4 (2.5)	
NA	22	5	17	
TMB				
High (>10 mutations/Mb)	59 (24.8)	10 (15.6)	49 (28.2)	0.06
Low (≤10 mutations/Mb)	179 (75.2)	54 (84.4)	125 (71.8)	
NA	2		2	
AS				
<4	59 (25.2)	11 (17.2)	48 (28.2)	0.22
4–24	159 (68)	48 (75)	111 (65.3)	
>24	16 (6.8)	5 (7.8)	11 (6.5)	
NA	6	0	6	
FGA				
<0.08	63 (26.5)	7 (10.9)	56 (32.2)	0.0008
>0.08	175 (73.5)	57 (89.1)	118 (67.8)	
NA	2	0	2	

CIN: chromosomal instability, MSI: microsatellite instability, GS: genomically stable, EBV: Epstein–Barr virus, POLE: polymerase epsilon, TMB: tumor mutation burden, AS: aneuploidy score, FGA: fragment genome altered, NA: not available.

## Data Availability

Beyond the data contained in the manuscript, no data is available.

## Supplementary Materials

The following supporting information can be downloaded at: https://www.mdpi.com/article/10.3390/jcm13247635/s1, Table S1. Clinical characteristics of the entire TCGA gastric cancer cohort and of the groups with no CDX2 induction (mRNA expression z score relative to all samples <−1) and with CDX2 induction (mRNA expression z score relative to all samples >0). Other histologies include signet ring, tubular, papillary and mucinous; Table S2. Genomic characteristics of the entire TCGA (The Cancer Genome Atlas) gastric cohort and of the groups with no CDX2 induction (mRNA expression z score relative to all samples <−1) and CDX2 induced (mRNA expression z score relative to all samples >0; Table S3. Mutations in DNA Damage Response (DDR) genes in samples with and without CDX2 mRNA suppression from the TCGA stomach cancer cohort; Table S4. Mutations in epigenetic modifiers in samples with and without CDX2 mRNA suppression from the TCGA stomach cancer cohort; Table S5. Mutations in receptor tyrosine kinases in samples with and without CDX2 mRNA suppression from the TCGA stomach cancer cohort.
