# Supplementary material for: Gastric Adenocarcinomas with CDX2 Induction Show Higher Frequency of TP53 and KMT2B Mutations and MYC Amplifications but Similar Survival Compared with Cancers with No CDX2 Induction
Source: J Clin Med. 2024 Dec 15;13(24):7635. doi: 10.3390/jcm13247635 (PMC11727917; doi:10.3390/jcm13247635)
Supplement: Supplementary file 1 [file jcm-13-07635-s001.zip › jcm-3337727-supplementary.pdf]

Supplementary Materials

Table S1. Clinical characteristics of the entire TCGA gastric cancer cohort and of the groups with no CDX2 induction (mRNA expression z score relative to all samples <-1) and with CDX2 induction (mRNA expression z score relative to all samples >0). Other histologies include signet ring, tubular, papillary and mucinous.

	**All patients in the 2 groups (*n* = 302) (%)**	**CDX2 not induced (*n* = 62) (%)**	**CDX2 induced (*n* = 240) (%)**	***p*-Value**
Age (mean)	65.4± 10.4	61.9± 10.3	66.5± 10.3	0.001
Early onset (≤50 years-old)				
yes	22 (7.4)	8 (12.9)	14 (5.9)	0.09
no	277 (92.6)	54 (87.1)	223 (94.1)	
NA	3		3	
Sex				
Male	202 (66.9)	45 (72.6)	157 (65.4)	0.36
Female	100 (33.1)	17 (27.4)	83 (34.6)	
Histology				
Intestinal/NOS adenocarcinoma	164 (54.3)	32 (51.6)	132 (55)	0.02
Diffuse	42 (13.9)	15 (24.2)	27 (11.2)	
Other	96 (31.8)	15 (24.2)	81 (33.8)	
Grade				
1-2	123 (41.6)	13 (21.7)	110 (46.6)	0.0004
3	173 (58.4)	47 (78.3)	126 (53.4)	
NA	6	2	4	
Stage				
I	48 (16.5)	13 (22)	35 (15.1)	0.29
II	91 (31.3)	14 (23.7)	77 (33.2)	
III	127 (43.6)	25 (42.4)	102 (44)	
IV	25 (8.6)	7 (11.9)	18 (7.7)	
NA	11	3	8	

NA: Not available, NOS: Not otherwise specified.

Table S2. Genomic characteristics of the entire TCGA (The Cancer Genome Atlas) gastric cohort and of the groups with no CDX2 induction (mRNA expression z score relative to all samples <-1) and CDX2 induced (mRNA expression z score relative to all samples >0

	**All patients in the 2 groups (*n* = 302) (%)**	**CDX2 not induced (*n* = 62) (%)**	**CDX2 induced (*n* = 240) (%)**	***p*-Value**
Genomic category				
CIN	163 (59.1)	25 (43.1)	138 (63.3)	<0.0001
GS	33 (12)	8 (13.8)	25 (11.5)	
MSI	55 (19.9)	8 (13.8)	47 (21.5)	
EBV	20 (7.2)	17 (29.3)	3 (1.4)	
POLE	5 (1.8)	0	5 (2.3)	
NA	26	4	22	
TMB				
High (>10 mutations/Mb)	69 (23)	10 (16.1)	59 (24.8)	0.17
Low (≤10 mutations/Mb)	231 (77)	52 (83.9)	179 (75.2)	
NA	2		2	
AS				
<4	100 (34)	18 (30)	82 (35.1)	0.61
4-24	175 (59.5)	39 (65)	136 (58.1)	
>24	19 (6.5)	3 (5)	16 (6.8)	
NA	8	2	6	
FGA				
< 0.08	84 (28)	21 (33.9)	63 (26.5)	0.26
> 0.08	216 (72)	41 (66.1)	175 (73.5)	
NA	2	0	2	

CIN: Chromosomal Instability, MSI: Microsatellite Instability, GS: Genomically Stable, EBV: Epstein-Barr Virus, POLE: Polymerase epsilon, TMB: Tumor Mutation Burden, AS: Aneuploidy Score, FGA: Fragment Genome Altered, NA: Not available.

Table S3. Mutations in DNA Damage Response (DDR) genes in samples with and without CDX2 mRNA suppression from the TCGA stomach cancer cohort.

Gene	All patients in the 2 groups (%)	CDX2 not induced (%)	CDX2 induced (%)	*p*-Value
BRCA1	11 (3.6)	3 (4,8)	8 (3.4)	0.7
BRCA2	23 (7.6)	5 (8.1)	18 (7.6)	1
PALB2	7 (2.3)	0	7 (2.9)	0.35
RAD51	1 (0.3)	0	1 (0.4)	1
RAD51B	0	0	0	1
RAD51C	1 (0.3)	0	1 (0.4)	1
RAD51D	1 (0.3)	0	1 (0.4)	1
RAD50	5 (1.6)	0	5 (2.1)	0.58
XRCC2	3 (1)	0	3 (1.3)	1
ATM	30 (10)	6 (9.7)	24 (10.1)	1
ATR	17 (5.6)	5 (8.1)	12 (5)	0.35
BRIP1	4 (1.3)	0	4 (1.7)	0.58
NBN	10 (3.3)	1 (1.6)	9 (3.8)	0.69
MRE11	6 (2)	1 (1.6)	5 (2.1)	1
CHEK1	5 (1.6)	0	5 (2.1)	0.58
CHEK2	6 (2)	0	6 (2.5)	0.35
BARD1	13 (4.3)	1 (1.6)	12 (5)	0.31
BAP1	9 (3)	1 (1.6)	8 (3.4)	0.69
POLQ	21 (7)	2 (3.2)	19 (8)	0.26
CDK12	11 (3.6)	1 (1.6)	10 (4.2)	0.46

Table S4. Mutations in epigenetic modifiers in samples with and without CDX2 mRNA suppression from the TCGA stomach cancer cohort.

**Gene**	**All patients in the 2 groups (%)**	**CDX2 not induced (%)**	**CDX2 induced (%)**	***p*-Value**
ARID2	26 (8.6)	8 (12.9)	18 (7.6)	0.2
ARID1A	78 (25.8)	19 (30.6)	59 (24.8)	0.41
ARID1B	19 (6.3)	5 (8.1)	14 (5.9)	0.55
ARID5B	12 (3.9)	1 (1.6)	11 (4.6)	0.47
KMT2C	47 (15.6)	12 (19.4)	35 (14.7)	0.43
KMT2D	51 (16.9)	13 (21)	38 (16)	0.34
KMT2A	32 (10.6)	3 (4.8)	29 (12.2)	0.1
KMT2B	32 (10.6)	2 (3.2)	30 (12.6)	0.03
DNMT3A	9 (3)	2 (3.2)	7 (2.9)	1
DNMT1	10 (3.3)	1 (1.6)	9 (3.8)	0.69
DNMT3B	8 (2.6)	2 (3.2)	6 (2.5)	0.67
KDM5C	9 (3)	2 (3.2)	7 (2.9)	1
KDM6A	11 (3.6)	1 (1.6)	10 (4.2)	0.47
KDM5A	14 (4.6)	3 (4.8)	11 (4.6)	1
EP300	15 (5)	2 (3.2)	13 (5.5)	0.74
CREBBP	31 (10.3)	4 (6.5)	27 (11.3)	0.35
SETD2	14 (4.6)	2 (3.2)	12 (5)	0.74
SMARCA2	15 (5)	1 (1.6)	14 (5.9)	0.32
SMARCA4	16 (5.3)	0	16 (6.7)	0.05
SMARCB1	8 (2.6)	0	8 (3.4)	0.21

Table S5. Mutations in receptor tyrosine kinases in samples with and without CDX2 mRNA suppression from the TCGA stomach cancer cohort.

Gene	All patients in the 2 groups (%)	CDX2 not induced (%)	CDX2 induced (%)	*p*-Value
EGFR	15 (5)	1 (1.6)	14 (5.9)	0.32
ERBB2	19 (6.3)	3 (4.8)	16 (6.7)	1
ERBB3	30 (9.9)	4 (6.5)	26 (10.9)	0.35
ERBB4	36 (11.9)	5 (8.1)	31 (13)	0.31
FGFR1	9 (3)	1 (1.6)	8 (3.4)	0.69
FGFR2	11 (3.6)	3 (4.8)	8 (3.4)	0.7
FGFR3	7 (2.3)	0	7 (2.9)	0.35
FGFR4	12 (3.6)	3 (4.8)	9 (3.8)	0.71
PDGFRA	10 (3.3)	1 (1.6)	9 (3.8)	0.69
PDGFRB	13(4.3)	1 (1.6)	12 (5)	0.31
NTRK1	9 (3)	3 (4,6)	6 (2.5)	0.39
NTRK2	13 (4.3)	0	13 (5.5)	0.07
NTRK3	13 (4.3)	2 (3.2)	11 (4.6)	1
ALK	13 (4,3)	1 (1.6)	12 (5)	0.31
ROS1	17 (5.6)	1 (1.6)	16 (6.7)	0.21
MET	6 (2)	1 (1.6)	5 (2.1)	1
RET	10 (3.3)	0	10 (4.2)	0.22
INSR	10 (3.3)	1 (1.6)	9 (3.8)	0.69
IGF1R	14 (4.6)	1 (1.6)	13 (5.5)	0.31
EPHA1	12 (4)	2 (3.2)	10 (4.2)	1
EPHB1	20 (6.6)	4 (6.5)	16 (6.7)	1
